# Causal relationship between gut microbiota and androgenetic alopecia: A Mendelian randomization study

**DOI:** 10.1097/MD.0000000000041106

**Published:** 2024-12-27

**Authors:** Jinyue Liu, Wenrong Luo, Zheyuan Hu, Xiaohai Zhu, Lie Zhu

**Affiliations:** a Department of Burns and Plastic Surgery, Changzheng Hospital, The Second Affiliated Hospital of Naval Medical University, Shanghai, China.

**Keywords:** AGA, gut microbiota, Mendelian randomization, SNPs

## Abstract

Recent studies have found a strong correlation between gut microbiota and the risk of skin diseases and proposed a “gut-skin axis.” Androgenetic alopecia (AGA) is the most common type of alopecia, and androgen plays an important role in its pathogenesis. It has been found that the gut microbiome is closely related to androgens; however, whether this relationship is causal or merely coincidental remains uncertain. To address this issue, Mendelian randomization (MR) analysis was performed to explore the association between gut microbiota and AGA. Genome-wide association studies (GWAS) have compiled summary statistics of the gut microbiota, including 211 taxa (131 genera, 35 families, 20 orders, 16 classes, and 9 phyla), with data from MiBioGen’s comprehensive study. We collected genetic associations with AGA from the IEU OpenGWAS project. We performed MR Analyses to assess the causal relationship between the genetically predicted gut microbiota and AGA. In order to verify the reliability of the findings, we systematically performed sensitivity analyses and heterogeneity tests and performed a heterogeneity test. MR Analysis provides important evidence for the causal relationship between genetically predicted gut microbiota and AGA. *Lachnospiraceae* UCG008 (OR = 0.939, 95%CI 0.175–0.775, *P* < .01), *Oxalobacte* (OR = 0.932, 95%CI 0.896–0.969, *P* < .01) would reduce the risk of AGA. *Eubacterium rectale* group (OR = 1.102, 95%CI 1.025–1.186, *P* < .01), *Roseburia* (OR = 1.183, 95%CI 1.048–1.336, *P* < .01) would increase the risk of AGA. Further sensitivity and heterogeneity analyses confirmed the robustness of these results. The results of this study indicate that there is a potential genetic susceptibility between gut microbiota and AGA, and screen out protective and risk factors. These results provide a theoretical basis for the prevention and treatment of AGA by regulating gut microbiota.

## 1. Introduction

Androgenetic alopecia (AGA), the most common type of hair loss, is characterized by progressive terminal hair loss after puberty. By 70 years of age, at least 80% of men and 50% of women develop the disease, and the incidence increases with age.^[1]^ AGA, as the name suggests, its pathogenesis is mainly derived from an overreaction to androgens. In AGA, the time of hair follicle in the growth phase is shortened and “miniaturized,” resulting in abnormally short and thin hair shafts, followed by the gradual transformation of normal hair to intermediate hair and vellus hair, and finally the gradual thinning and alopecia of AGA patients.^[2]^ This premature alopecia can have a significant negative impact on the patient’s self-image and can even cause psychological disorders such as anxiety, anxiety, and depression.^[3]^ Existing evidence suggests that the development and progression of AGA depends on the interaction between endocrine factors and genetic susceptibility. However, the exact pathogenesis of AGA is still unclear, which limits the existing treatments.^[4]^ The gut microbiota is a major regulator of androgen metabolism in intestinal contents. This also suggests that the imbalance of gut microbiota may be one of the important causes of AGA.

Gut microbiota is a complex combination of multiple microorganisms, which is closely related to human health and disease. Metabolic disorders, composition imbalance and dysfunction of intestinal microbiota can cause various diseases.^[5]^ Increasing evidence suggested that alterations in gut microbiota composition are closely related to abnormal androgen metabolism, and many scholars have proposed the hypothesis of “the gut-testis axis.”^[6]^ Oral administration of *lactic acid bacteria* or *lactobacillus reuteri* could increase serum testosterone levels and achieve testicular enlargement.^[7,8]^ It can be seen that gut microbiota is closely related to the metabolism of human androgens, and androgens play an important role in the pathogenesis of AGA. Therefore, gut microbiota may be associated with AGA through the “gut-testis axis.” However, the causal relationship between gut microbes and AGA needs to be further studied.

Randomized controlled trials (RCTs) are the gold standard for traditional methods to infer the causal relationship between outcome and exposure. However, in some cases, due to ethical requirements or financial problems, it is very difficult to fully implement RCTs. Mendelian randomization (MR) studies perfectly avoid these difficulties. MR studies uses genetic variants as instrumental variables to assess causality from an exposure factor to an outcome and can therefore be used as an alternative to RCTs. According to Mendelian law of inheritance, in the process of meiosis, parental alleles will be randomly assigned to the offspring, that is, random genetic variants will appear in this process, and the selected genetic variants will not be affected by the complex social and economic factors after birth. The MR studies are similar in concept to RCTs, with patients randomly assigned to an exposure or control group. The difference is that MR studies are assigned to patients on the basis of their genotype. Genetic variants were used as instrumental variables (IVs) to infer causal effects between exposure and outcome. As more and more genome-wide association studies (GWAS) data are shared by researchers, a large number of reliable genetic variants have been obtained in MR studies.

In this study, we used a 2-sample MR approach, with GWAS data of gut microbiota as the exposure data and GWAS data of AGA as the outcome data, to examine the causal relationship between gut microbiota and AGA. Therefore, 3 key hypotheses were formulated in this study: there is a significant association between IVs and gut microbiota; IVs was not associated with all confounding factors of gut microbiota-AGA; IVs could not directly affect the results, but only indirectly through the association with gut microbiota.

## 2. Materials and methods

All analyses in this study were performed using R software, and MR study was mainly performed relying on 2-sample MR in the R package.

### 2.1. Data sources

**Figure 1.**
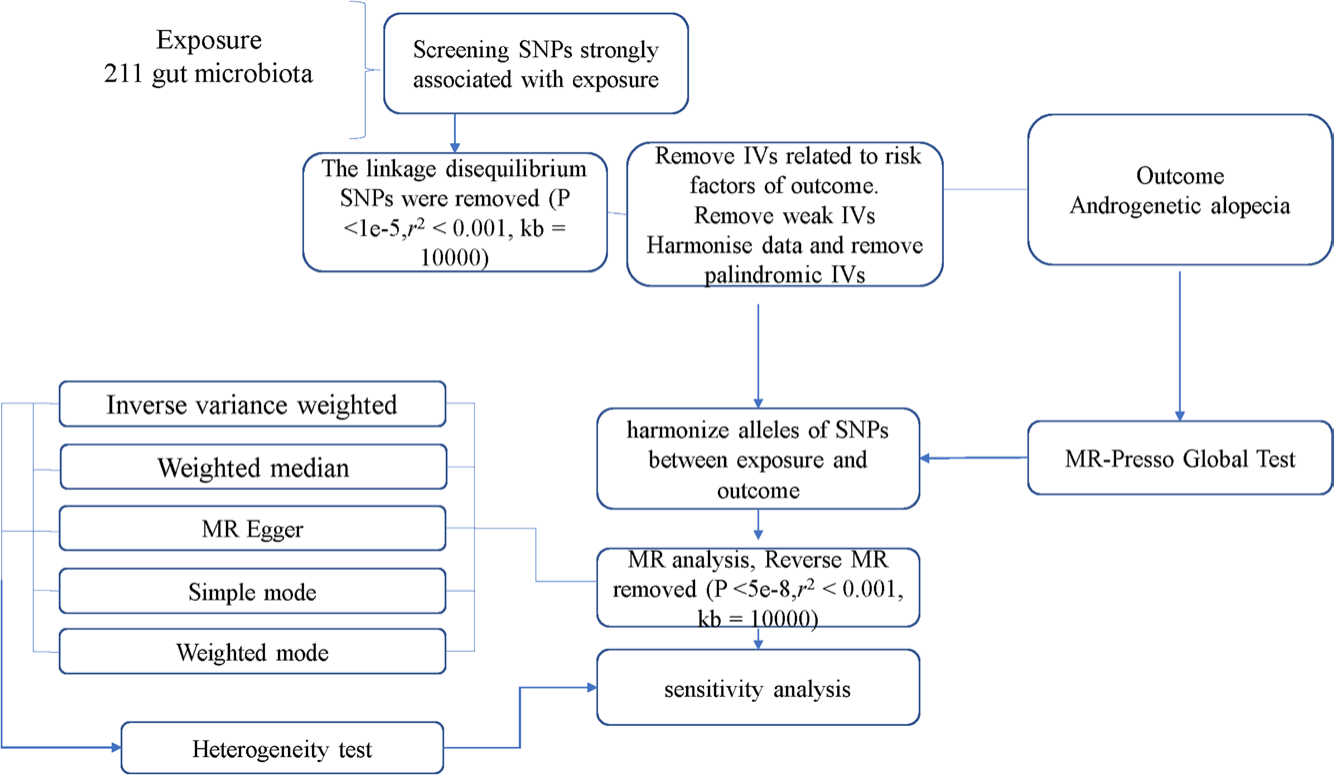
The overall flow of this study. IVs = instrumental variables, MR = Mendelian randomization, SNPs = single nucleotide polymorphisms.

The GWAS data of gut microbiota in this study were collected from the MiBioGen consortium (https://mibiogen.gcc.rug.nl/), which analyzed the genome-wide genotype and 16S fecal microbiome data from 18,340 individuals from 24 countries, including the United States, Canada, Israel, South Korea, the United Kingdom, etc, with a total of 211 taxa (131 genera, 35 families, 20 orders, 16 classes and 9 phyla).^[9]^ GWAS data for AGA were obtained from the IEU OpenGWAS project (GWAS Catalog [ebi.ac.uk]). Figure 1 briefly presents the overall flow of this study.

### 2.2. Selection of IVs

**Figure 2.**
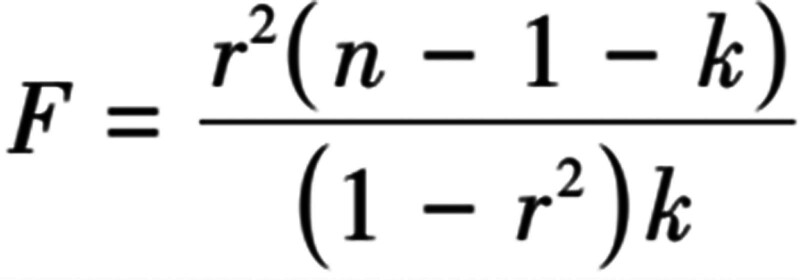
Formula for the calculation of *F* statistic.

To ensure the accuracy and validity of the causal relationship between gut microbiota and AGA, the following quality control steps were used to select the best IVs: At genome-wide statistical significance threshold (*P* < 1.0 × 10^−5^), single nucleotide polymorphisms (SNPs) associated with each genus were selected as potential IVs; SNPs in each bacterial taxon were pooled and only independent SNPs were retained. The linkage disequilibrium parameter *r*^2^ was set to <0.001, and the genetic distance was 10,000 kb, and the SNPs with the smallest *P* value were selected to ensure the independence of IVs and exclude the influence of linkage disequilibrium on the results; The data were preprocessed to keep the effect allele and effect size consistent, and the palindromic SNPs (A/T and A/C alleles) were excluded; Heterogeneity test, pleiotropy test and leave-one-out test were used to evaluate the stability of the results, and the effect of pleiotropy was eliminated by removing outliers; The *F* statistic of selected SNPs was evaluated using the following equation (Fig. 2): *r*^2^ represents the extent to which exposure was explained by IVs, *n* represents the sample number of exposure GWAS studies, *k* represents the number of SNPs. *F* statistic represents the variance, goodness of fit and significance of regression models between 2 or more groups, and can evaluate the weak instrumental variable effect.^[10]^ *F* statistic < 10 indicating that it is a weak instrumental variable and was excluded.^[11]^

### 2.3. Statistical analysis

This 2-sample MR study was performed to estimate the causal effects between gut microbiota and AGA using inverse variance weighting (IVW),^[12]^ MR-Egger test,^[13]^ weighted median (WME),^[14]^ weighted mode^[15]^ and simple mode methods.^[16]^ Each statistical method operated under an independent assumption model: IVW assumed no horizontal pleiotropy; MR-Egger assumed pleiotropy in more than half of the SNPs; WME assumed pleiotropy in less than half of the SNPs. The inclusion of simple mode aids in dissecting the experimental effect and determining the significance of treatment effect by comparing it with actual observations. Amongst these methods, IVW demonstrated higher accuracy compared to others. Therefore, this study primarily relied on results obtained from IVW method while supplementing them with findings from other approaches. To assess result stability, *Q* test^[17]^ and MR-Egger test were conducted using IVW method to examine potential heterogeneity and pleiotropy respectively. Furthermore, Leave-one-out analysis was used to explore whether a single SNP affected the causal effect, that is, sensitivity analysis was performed on the results.

#### 2.3.1. Statement of ethics

This study does not involve human or animal experiments, all data are from public databases, and no ethical review is required.

### 2.4. Reverse MR study

We also performed an additional MR study to investigate reverse causality between AGA as the exposure factor and gut microbial signature as the outcome, at genome-wide statistical significance thresholds (*P* < 5.0 × 10^−8^). The reverse MR study procedure was the same as above.

## 3. Results

### 3.1. Instrumental variables

**Figure 3.**
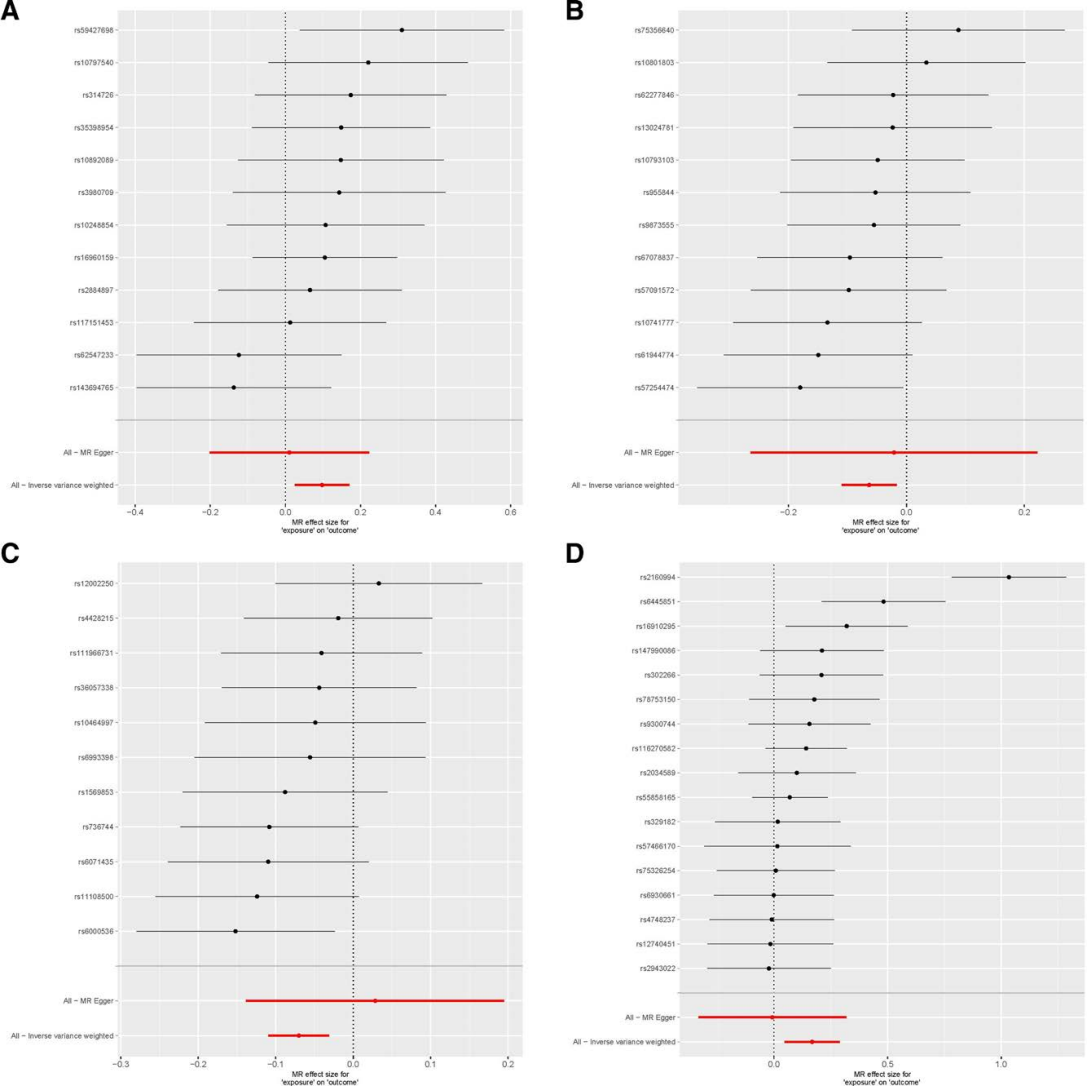
Forest plots for MR estimates of the causal effect of gut microbiota in AGA using IVW. (A) *Eubacterium rectale* group. (B) *Lachspirillaceae* UCG008. (C) *Oxalobacter*. (D) *Roseburia*. AGA = androgenetic alopecia, IVW = inverse variance weighting, MR = Mendelian randomization.

In this study, GWAS data of 211 gut microbiotas were selected as study exposures, including 9 phyla, 16 classes, 20 orders, 35 families, and 131 genera. *P* < 1.0 × 10^−5^ and the threshold of linkage disequilibrium analysis were used to obtain different numbers of SNPs. *F* statistics are all >10, indicating no weak instrument bias. The results were mainly analyzed by IVW, as detailed in Table 1 and Figure 3.

**Table 1:** Results of MR study between gut microbiota and AGA.

Categories	Traits	SNP	β	SE	OR	95%CI	*P*	Pleiotropy			Heterogeneity		
								Egger_ intercept	SE	*P*	Method	Cochran’s *Q*	*P*
Genus	*Eubacterium rectale* group	12	0.097	0.037	1.102	1.025~1.186	.009	0.006	0.007	.413	IVW	10.102	.521
Genus	*Lachnospiraceae* UCG008	12	−0.063	0.024	0.939	0.896~0.984	.007	−0.004	0.013	.735	IVW	8.431	.674
Genus	*Oxalobacter*	11	−0.070	0.020	0.932	0.896~0.969	<.001	−0.014	0.012	.263	IVW	6.51	.771
Genus	*Roseburia*	17	0.168	0.062	1.183	1.048~1.336	.006	−0.074	0.097	.462	IVW	9.954	<.001

AGA = androgenetic alopecia, IVW = inverse variance weighting, MR = Mendelian randomization, SNP = single nucleotide polymorphism.

### 3.2. 2-sample MR study

In this study, a 2-sample MR study was used to analyze the causal relationship between the relative abundance of gut microbes and AGA at the level of phylum, class, order, family, and genus. The analysis results were mainly based on IVW method and supplemented by other methods.

**Figure 4.**
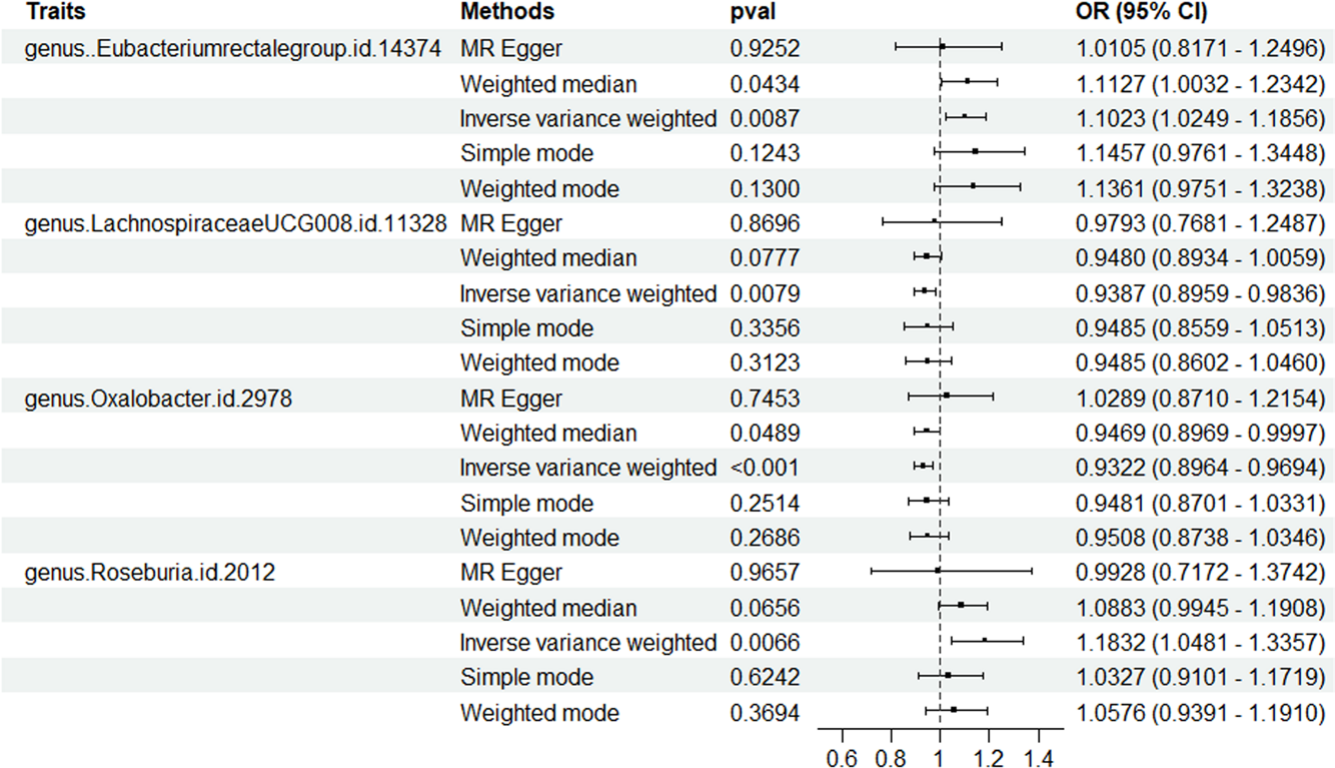
Forest plot for MR estimates of the causal effect of gut microbiota in AGA using MR Egger, WME, IVW, simple mode and weighted median. AGA = androgenetic alopecia, IVW = inverse variance weighting, MR = Mendelian randomization, WME = weighted median.

**Figure 5.**
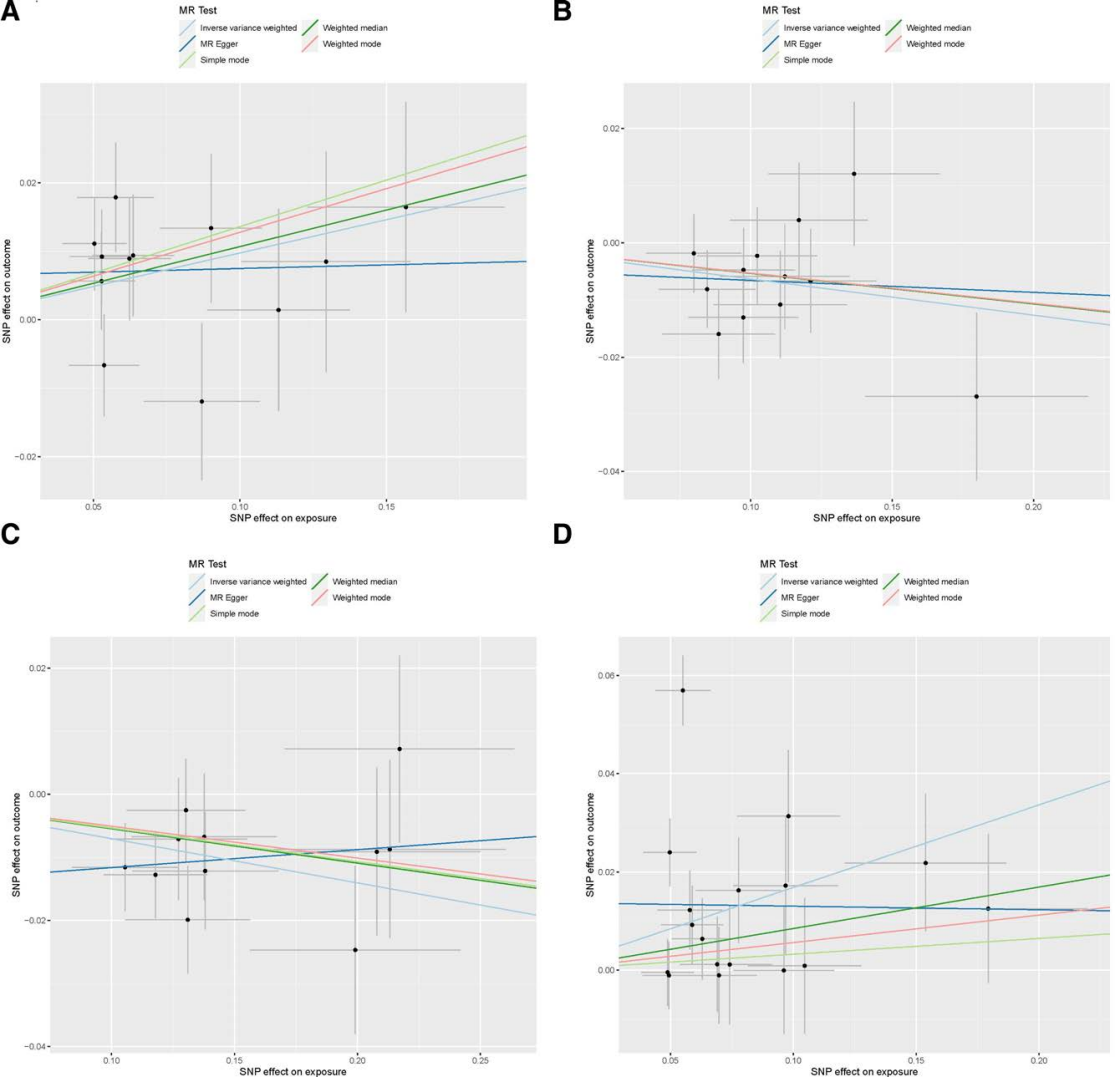
Scatter plot for MR estimates of the causal effect of gut microbiota in AGA using MR Egger, WME, IVW, simple mode and weighted median. (A) *Eubacterium rectale* group. (B) *Lachspirillaceae* UCG008. (C) *Oxalobacter*. (D) *Roseburia*. AGA = androgenetic alopecia, IVW = inverse variance weighting, MR = Mendelian randomization, WME = weighted median.

As shown in Figure 4, among different methods, except IVW, the other 4 results were not significant (*P* > .05), indicating that the results of this study were less stable. Figure 5 shows that the MR study with IVW can be used to obtain the results that the *Lachspirillaceae* UCG008 (OR = 0.939, 95%CI 0.175–0.775, *P* < .01), *Oxalobacter* (OR = 0.932, 95%CI 0.896–0.969, *P* < .01) could reduce the risk of AGA. *Eubacterium rectosum* (OR = 1.102, 95%CI 1.025–1.186, *P* < .01), *Roseburia* (OR = 1.183, 95%CI 1.048–1.336, *P* < .01) could increase the risk of AGA. The detailed results are shown in Table 1.

### 3.3. Quality control

In order to further test the stability and reliability of the results, the quality of the included SNPs was controlled by heterogeneity test, pleiotropy test and sensitivity analysis.

#### 3.3.1. Leave-one-out analysis

**Figure 6.**
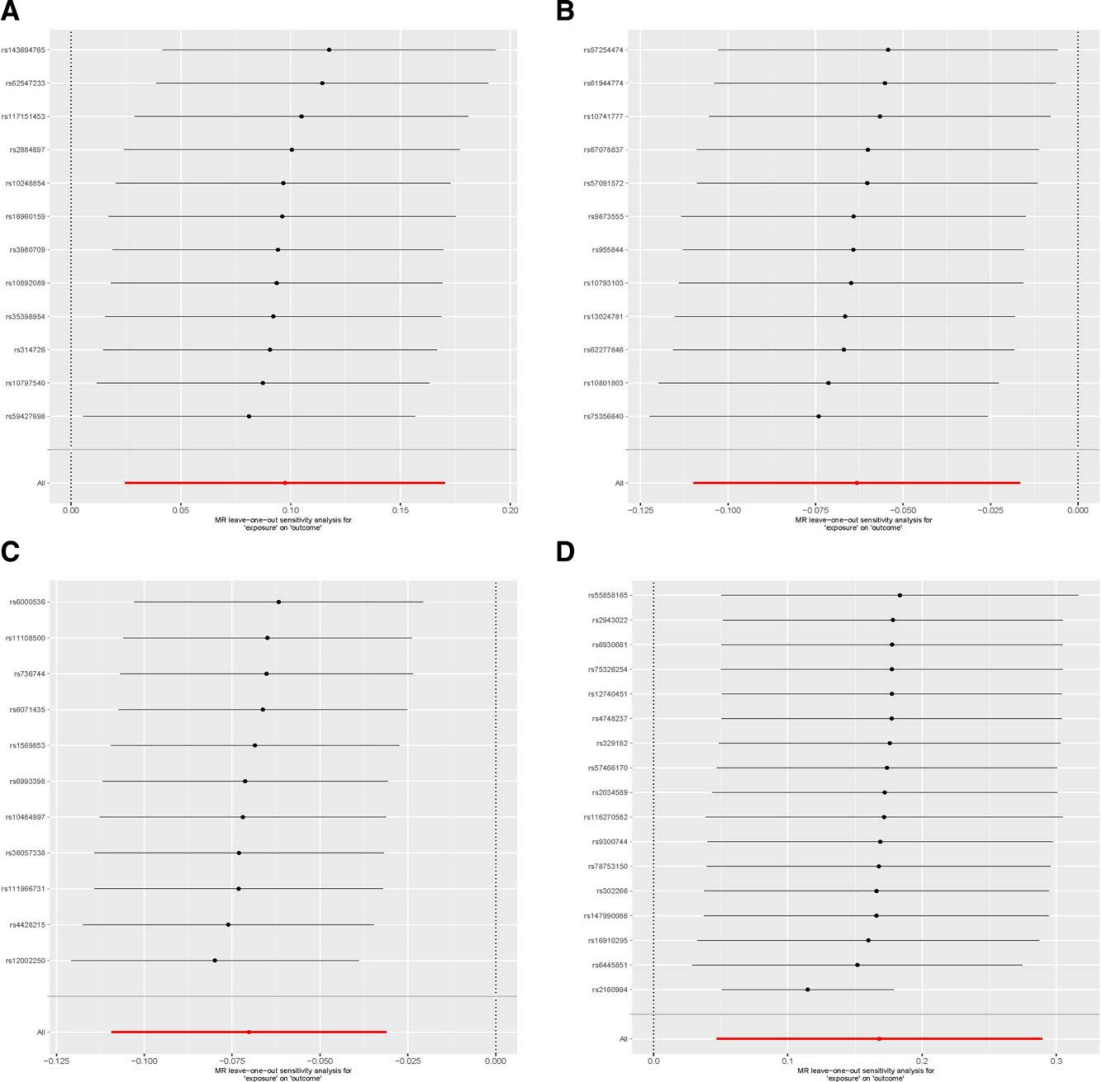
Forest plot for MR estimates of the causal effect of gut microbiota in AGA using leave-one-out analysis. (A) *Eubacterium rectale* group. (B) *Lachspirillaceae* UCG008. (C) *Oxalobacter*. (D) *Roseburia*. AGA = androgenetic alopecia, MR = Mendelian randomization.

The results of leave-one-out analysis showed that the effect size of the IVs included was close to the total effect size, and no SNPs were found to have great impact on the causal association estimate (Fig. 6). The above sensitivity analysis showed that the results of our study were stable and reliable.

#### 3.3.2. Heterogeneity test and pleiotropy test

Except for *Roseburia*, the results of heterogeneity test and pleiotropy test of the included SNPs were all *P* > .05, indicating that the effects of heterogeneity and pleiotropy on the study results were not needed to be considered. The detailed results are shown in Table 1. Although *Roseburia*’s heterogeneity test was *P* < .05, indicating that there may be some heterogeneity, it did not affect the IVW results. Therefore, our conclusion is still reliable. The MR-Egger Intercept can be used to detect the existence of horizontal pleiotropy. All *P* values of MR-Egger intercept in this study are >.05, which can be considered that there is no horizontal pleiotropy in this study. This indicated that the relationship between IVs and outcomes was not affected by potential confounding factors. The effect of IVs on the outcome mainly occurs through direct effects rather than indirect pathways. The independence assumption and the exclusion restrictive assumption were verified by MR-Egger to exclude the influence of confounding factors and to determine the independence of IVs on the outcome.

### 3.4. Results of the reverse MR study

We performed reverse MR study using the GWAS data of AGA as the exposure and the GWAS data of gut microbiome as the outcome, at genome-wide statistical significance thresholds (*P* < 5.0 × 10^−8^), after removing linkage disequilibrium and palindromic SNPs, we could not obtain SNPs of exposure in the outcome, that is, there was no evidence of causal effect between AGA and the identified gut microbiota.

## 4. Discussion

In this study, we used GWAS data of gut microbiota from public databases as exposure and GWAS data of AGA as outcome to analyze the causal relationship between 211 gut microbiota and AGA using a 2-sample MR study. *Lachnospiraceae* UCG008 and *Oxalobacter* in gut microbes were found to be protective factors for AGA, while *Eubacterium rectale* group and *Roseburia* were risk factors for AGA.

In many studies, *Lachnospiraceae* has been shown to produce beneficial metabolites in the host.^[18]^ Diseases including metabolic syndrome, obesity, diabetes, liver disease, inflammatory bowel disease and chronic kidney disease were found to be associated with changes in *Lachnospiraceae* abundance. However, it has not been reported that *Lachnospiraceae* is associated with AGA or androgens. We are the first to propose that the increased abundance of *Lachnospiraceae* UCG008 reduces the risk of AGA. Polycystic ovary syndrome (PCOS) is a common endocrine and metabolic disorder in women of reproductive age. It is characterized by ovulation dysfunction or loss and hyperandrogenism. The main clinical manifestations are irregular menstrual cycle, infertility, hirsutism and acne. Decreased *Oxalobacter* abundance was found in the PCOS model, and the abnormal gut microbiome was earlier than the onset of clinical symptoms of PCOS.^[19]^ In addition, it was found that serum androgen levels were negatively correlated with *Eubacterium rectale* group abundance in PCOS patients.^[20]^ Reduced *Oxalobacter* abundance was found in the gut of some prostate cancer patients undergoing androgen deprivation therapy with medical or surgical castration.^[21]^ Meanwhile, a significant correlation between androgen levels and *Oxalobacter* abundance was also found in a case-control trial.^[22]^ All the above reports show that gut microbiota is closely related to androgens, but it is not enough to explain whether there is a causal relationship between gut microbiota and androgens or whether there is a causal relationship between gut microbiota and AGA. AGA is a multifactorial disease caused by genetic factors, hormonal dysregulation, environmental and systemic factors, and aging.^[23]^ Androgens play an important role in the development of AGA. During sexual maturation, androgens promote hair follicle growth, and androgen binding to androgen receptors on hair follicles promotes proliferation of dermal papilla cells in androgen-sensitive sites such as the axilla, pubis, face, chest, and limbs. However, at a later age, the effect of androgens on genetically susceptible hair follicles is instead detrimental, promoting their miniaturization and ultimately causing alopecia.^[24]^ Therefore, these studies provided a theoretical basis for the effect of microbiota on androgen metabolism and the occurrence of androgenetic alopecia.

We carried out this MR study and obtained that gut microbiota dysbiosis is one of the causes of AGA, in which *Lachnospiraceae* UCG008 and *Oxalobacter* are protective factors of AGA, *Eubacterium rectale* group and *Roseburia* were risk factors for AGA, and AGA was not the cause of gut microbiota dysregulation. The gut microbiota may affect the onset of AGA by regulating intestinal androgen metabolism, which is consistent with the hypothesis of “gut-testis axis” proposed by previous researchers.^[6]^

Based on the known association between gut microbiota and AGA, our study further investigated the causal relationship by a 2-sample MR study. The strengths of this study include the following 2 points: The MR study was used to effectively avoid confounding factors, and multiple sensitivity analyses were used to confirm the robustness of our conclusions; The causal relationship identified may provide candidate bacteria for further mechanistic studies and probiotic development.

Of course, this study also has some limitations. First, we only used the GWAS data of the European population for analysis, and future studies on other populations are needed. Second, the abundance of gut microbiota included in the study was limited. Although the GWAS data we used was the gut microbiota sequencing study with the largest population cohort before writing, it is still necessary to more comprehensively investigate the relationship between gut microbiota and AGA as the research continues. Third, MR study is an efficient method for causality analysis, but animal experiments are still needed to verify it.

## 5. Conclusion

In summary, we evaluated the causal relationship between gut microbiota and AGA. The 2 bacteria had a negative causal relationship with AGA. There is a positive causal relationship between the 2 bacteria and AGA. Its mechanism of action may be by affecting androgen metabolism, leading to the pathogenesis of AGA, but it still needs to be further verified.

## Author contributions

**Conceptualization:** Lie Zhu.

**Data curation:** Xiaohai Zhu.

**Formal analysis:** Xiaohai Zhu.

**Funding acquisition:** Lie Zhu.

**Resources:** Zheyuan Hu.

**Software:** Wenrong Luo, Zheyuan Hu.

**Writing – original draft:** Jinyue Liu.

**Writing – review & editing:** Jinyue Liu, Wenrong Luo.
